# Cuba: Exploring the History of Admixture and the Genetic Basis of Pigmentation Using Autosomal and Uniparental Markers

**DOI:** 10.1371/journal.pgen.1004488

**Published:** 2014-07-24

**Authors:** Beatriz Marcheco-Teruel, Esteban J. Parra, Evelyn Fuentes-Smith, Antonio Salas, Henriette N. Buttenschøn, Ditte Demontis, María Torres-Español, Lilia C. Marín-Padrón, Enrique J. Gómez-Cabezas, Vanesa Álvarez-Iglesias, Ana Mosquera-Miguel, Antonio Martínez-Fuentes, Ángel Carracedo, Anders D. Børglum, Ole Mors

**Affiliations:** 1 National Centre of Medical Genetics, Medical University of Havana, La Habana, Cuba; 2 Department of Anthropology, University of Toronto at Mississauga, Mississauga, Ontario, Canada; 3 Unidade de Xenética, Departamento de Anatomía Patolóxica e Ciencias Forenses, and Instituto de Ciencias Forenses, Grupo de Medicina Xenómica (GMX), Facultade de Medicina, Universidade de Santiago de Compostela, Galicia, Spain; 4 Translational Neuropsychiatry Unit, Department of Clinical Medicine, Aarhus University, Aarhus, Denmark; 5 Centre for Integrative Sequencing (iSEQ), Aarhus University, Aarhus, Denmark; 6 The Lundbeck Foundation Initiative for Integrative Psychiatric Research, Aarhus University, Aarhus, Denmark; 7 Department of Biomedicine, Aarhus University, Aarhus, Denmark; 8 Centro Nacional de Genotipado (ISCIII), Nodo Santiago de Compostela, Santiago de Compostela, Spain; 9 Centro de Investigaciones Psicológicas y Sociológicas, La Habana, Cuba; 10 Departamento de Antropología, Facultad de Biología, Universidad de La Habana, La Habana, Cuba; 11 Center of Excellence in Genomic Medicine Research, King Abdulaziz University, Jeddah, Kingdom of Saudi Arabia; 12 Psychiatric Department, Aarhus University Hospital, Aarhus, Denmark; University of Washington, United States of America

## Abstract

We carried out an admixture analysis of a sample comprising 1,019 individuals from all the provinces of Cuba. We used a panel of 128 autosomal Ancestry Informative Markers (AIMs) to estimate the admixture proportions. We also characterized a number of haplogroup diagnostic markers in the mtDNA and Y-chromosome in order to evaluate admixture using uniparental markers. Finally, we analyzed the association of 16 single nucleotide polymorphisms (SNPs) with quantitative estimates of skin pigmentation. In the total sample, the average European, African and Native American contributions as estimated from autosomal AIMs were 72%, 20% and 8%, respectively. The Eastern provinces of Cuba showed relatively higher African and Native American contributions than the Western provinces. In particular, the highest proportion of African ancestry was observed in the provinces of Guantánamo (40%) and Santiago de Cuba (39%), and the highest proportion of Native American ancestry in Granma (15%), Holguín (12%) and Las Tunas (12%). We found evidence of substantial population stratification in the current Cuban population, emphasizing the need to control for the effects of population stratification in association studies including individuals from Cuba. The results of the analyses of uniparental markers were concordant with those observed in the autosomes. These geographic patterns in admixture proportions are fully consistent with historical and archaeological information. Additionally, we identified a sex-biased pattern in the process of gene flow, with a substantially higher European contribution from the paternal side, and higher Native American and African contributions from the maternal side. This sex-biased contribution was particularly evident for Native American ancestry. Finally, we observed that SNPs located in the genes *SLC24A5* and *SLC45A2* are strongly associated with melanin levels in the sample.

## Author Summary

Cuba is the largest island of the Greater Antilles and its most populous country. The post-Columbian history of the Caribbean has been marked by the encounter of people from different continents. Here, we present an admixture analysis of 1,019 individuals from all the provinces of Cuba, using autosomal, mtDNA and Y-chromosome markers. We also analyzed the association of 16 single nucleotide polymorphisms (SNPs) with quantitative estimates of skin pigmentation (melanin index). The highest proportions of African ancestry were observed in the Southeastern provinces of Santiago de Cuba and Guantánamo, and the highest proportions of Native American ancestry were found in the Eastern provinces of Granma, Holguín and Las Tunas. Similar geographic patterns were observed in the analyses of the uniparental markers. Additionally, by comparing the autosomal and uniparental admixture proportions, we identified a clear sex-biased pattern in the process of gene flow, with a substantially higher European contribution from the paternal side than the maternal side, and conversely higher Native American and African contributions from the maternal side than the paternal side. Finally, we observed that SNPs located in the genes *SLC24A5* and *SLC45A2* show a strong association with skin pigmentation in the sample.

## Introduction

The post-Columbian history of the Caribbean has been marked by the encounter of people from different continents. This is reflected in the gene pool of the present inhabitants of the Caribbean archipelago, as shown in recent studies using autosomal, mtDNA and Y-chromosome markers [1]–[7]. However, very few studies have focused on Cuba, the largest island of the Greater Antilles [8], [9].

Evidence of human habitation in Cuba goes back to approximately 7,000 years BP [10], [11]. It has been estimated that at the arrival of the Spaniards there were around 110,000 indigenous people living on the island [12]. At the time of contact there were two indigenous groups in Cuba. The Guanahatabey were hunter-gatherers living in western Cuba. They comprised approximately 10% of the indigenous Cuban population, spoke a non-Arawak language and have been considered to be the descendants of the earliest settlers of the island. The Taino were Arawak-speaking agriculturalists inhabiting the rest of the island, and comprised 90% of the indigenous population. The most accepted hypothesis is that both groups migrated from South America (lower Orinoco Valley) [11], [12]. However, North American (Florida) and Mesoamerican (Yucatan, Honduras and Nicaragua) migrations have also been postulated by some authors, particularly for the earliest settlers of the island [11], [12]. Within 50 years of the arrival of Columbus, the indigenous Cuban population had been decimated to a few thousand people. The Spaniards then started to relocate indigenous people from North America and Mesoamerica to Cuba, as well as enslaved Africans, primarily from the West Coast of Africa [9], [13]. It has been estimated that between 700,000 and 1,300,000 Africans were brought to Cuba during the slave trade period [14], [15]. Immigration from Spain took place throughout the colonial and post-colonial periods, until the first half of the 20^th^ century. Historical records indicate that most of the immigrants from Spain were male (60–85%), and that mixing between European males and indigenous and African females occurred since the early stages of the colonization of the island [12]. Therefore, the present genetic structure of the Cuban population has been shaped by the history of admixture between indigenous Americans, Europeans and Africans. Today, the Cuban census classifies the population into three categories: “Blancos” (“White”), “Mestizos” (“Mixed”) and “Negros” (“Black”) [16].

Here, we present an admixture analysis of a large sample comprising 1,019 individuals from the 16 provinces of Cuba. We used a panel of 128 Ancestry Informative Markers (AIMs) to estimate the admixture proportions. In addition to the AIMs, we also characterized a number of haplogroup diagnostic SNPs in the mtDNA and Y-chromosome in order to evaluate admixture using uniparental markers. Finally, we also evaluated the association of 16 single nucleotide polymorphisms (SNPs) with skin pigmentation.

This study is relevant from different points of view. Understanding the distribution of admixture proportions throughout Cuba is important from an anthropological perspective, and this is the most extensive effort carried out to date in terms of the size and representativeness of the sample. Additionally, the study of uniparental markers provides interesting evidence regarding the directionality of gene flow. The elucidation of admixture proportions is also of interest for future application of admixture mapping studies or genome-wide association studies in Cuba. Finally, we show that SNPs located in the genes *SLC24A5* and *SLC45A2* are strongly associated with melanin levels in the sample.

## Results

The average age of the participants was 49.35 years (minimum 18; maximum 95; SD 16.59) and 58% were female. The participants came from all the provinces of Cuba, primarily from urban areas (77% vs. 23% from rural areas). In terms of the self-reported census classification, 55% of the participants indicated to be “blanco”, 33% “mestizo” and 12% “negro” (Table 1).

**Table 1:** Demographic characteristics of the sample.

Categories		Absolute frequency	%
**Sex**	Female	590	58
	Male	429	42
**Census Category**	“Blanco”	560	55
	“Mestizo”	337	33
	“Negro”	122	12
**Age (years)**	15–19	17	1,7
	20–39	276	27,1
	40–49	253	24,8
	50–59	186	18,3
	60–69	152	14,9
	70–79	97	9,5
	80–84	24	2,4
	>85	14	1,4
**Province**	Pinar del Río (PR)	76	7.5
	Artemisa (AR)	37	3.6
	Mayabeque (MY)	33	3.2
	La Habana (LH)	94	9.2
	Matanzas (MT)	72	7.1
	Cienfuegos (CF)	45	4.4
	Villa Clara (VC)	95	9.3
	Sancti Spíritus (SS)	52	5.1
	Ciego de Ávila (CA)	48	4.7
	Camagüey (CG)	80	7.9
	Las Tunas (LT)	48	4.7
	Holguín (HG)	109	10.7
	Granma (GR)	70	6.9
	Santiago de Cuba (SC)	96	9.4
	Guantánamo (GT)	55	5.4
	Isla de la Juventud (IJ)	9	0.9
**Urban/Rural**	Urban	784	77
	Rural	235	23

### Distribution of autosomal admixture proportions in the total sample and stratified by provinces

**Figure 1:**
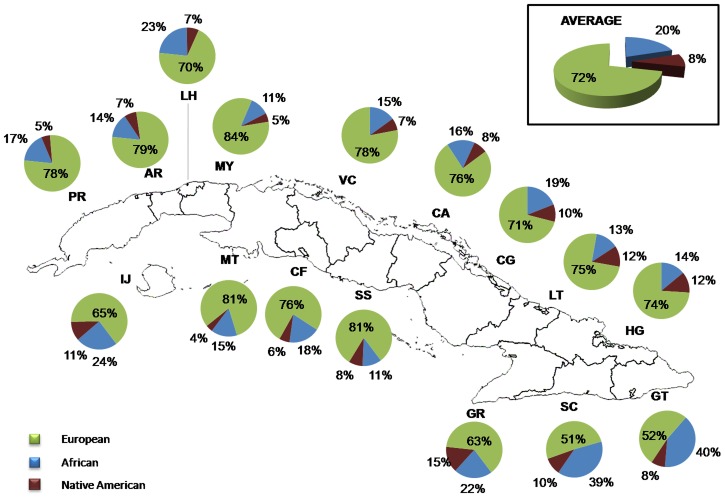
Distribution of ancestral contributions in the total sample and stratified by province as inferred from autosomal AIMs.

Estimates of admixture proportions were obtained with the program ADMIXMAP, using data from 128 AIMs. In the total sample, the average European, African and Native American contributions were 72% (range 4.3% to 98.2%), 20% (range 0.8% to 95.2%) and 8% (range 0.4% to 34%), respectively (Figure 1). By province, the average proportion of European ancestry ranged from 51% in Santiago de Cuba to 84% in Mayabeque, the average proportion of African ancestry ranged from 11% in Mayabeque and Sancti Spíritus to 40% in Guantánamo, and the average proportion of Native American ancestry from 4% in Matanzas to 15% in Granma (Figure 1). There are significant differences in admixture proportions between provinces (ANOVA: Africans *F* = 11.54, *P*<0.001; Native American *F* = 13.06, *P*<0.001). *Post-hoc* tests indicate that, in terms of African proportions, the differences are driven by the higher African proportions in the provinces of Santiago de Cuba (39%) and Guantánamo (40%), with respect to the other provinces (11% to 24%). With respect to the Native American contributions, a clear pattern is also present, with higher average contributions in the Eastern provinces, particularly Granma (15%), Las Tunas (12%) and Holguín (12%) than in the Western provinces.

### Relationship between census categories, melanin levels and individual ancestry proportions

As indicated above, 55% of the participants self-reported to be “blanco”, 33% “mestizo” and 12% “negro”. These proportions are similar to those based on the report of an external observer; there were discrepancies for only 65 out of the 1019 individuals. Several measures of concordance indicated excellent agreement between both classifications (Cohen's *kappa* = 0.8873 [17], Ciccheti-Allison's *kappa* = 0.9091 [18] and Fleiss-Cohen's *kappa* = 0.9345 [19]).

**Figure 2:**
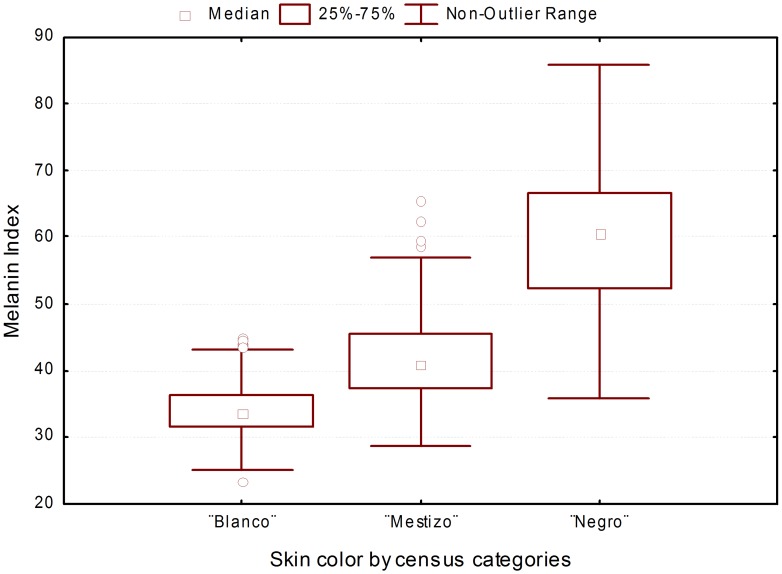
Distribution of melanin index stratified by census categories.

Age did not have a significant effect on melanin levels (M), measured quantitatively with the reflectometer (melanin index) [20], but there were significant differences in melanin index by sex (males M = 40.68±10.7; females M = 39.17±9.45; *P* = 0.015). The average melanin index of the total sample was 39.8, but there was a broad distribution of values, from 23.4 to 85.9. In individuals who self-reported to be “blanco”, the average melanin index was 34.06±3.70 (*mean ± SD*), in those who self-reported to be “mestizo” 41.69±6.29 and in those who self-reported to be “negro” 60.59±8.87 (Figure 2). The differences in melanin levels between census groups were significant (ANOVA with sex as a covariate: *F* = 4.30, *P*<0.001).

**Figure 3:**
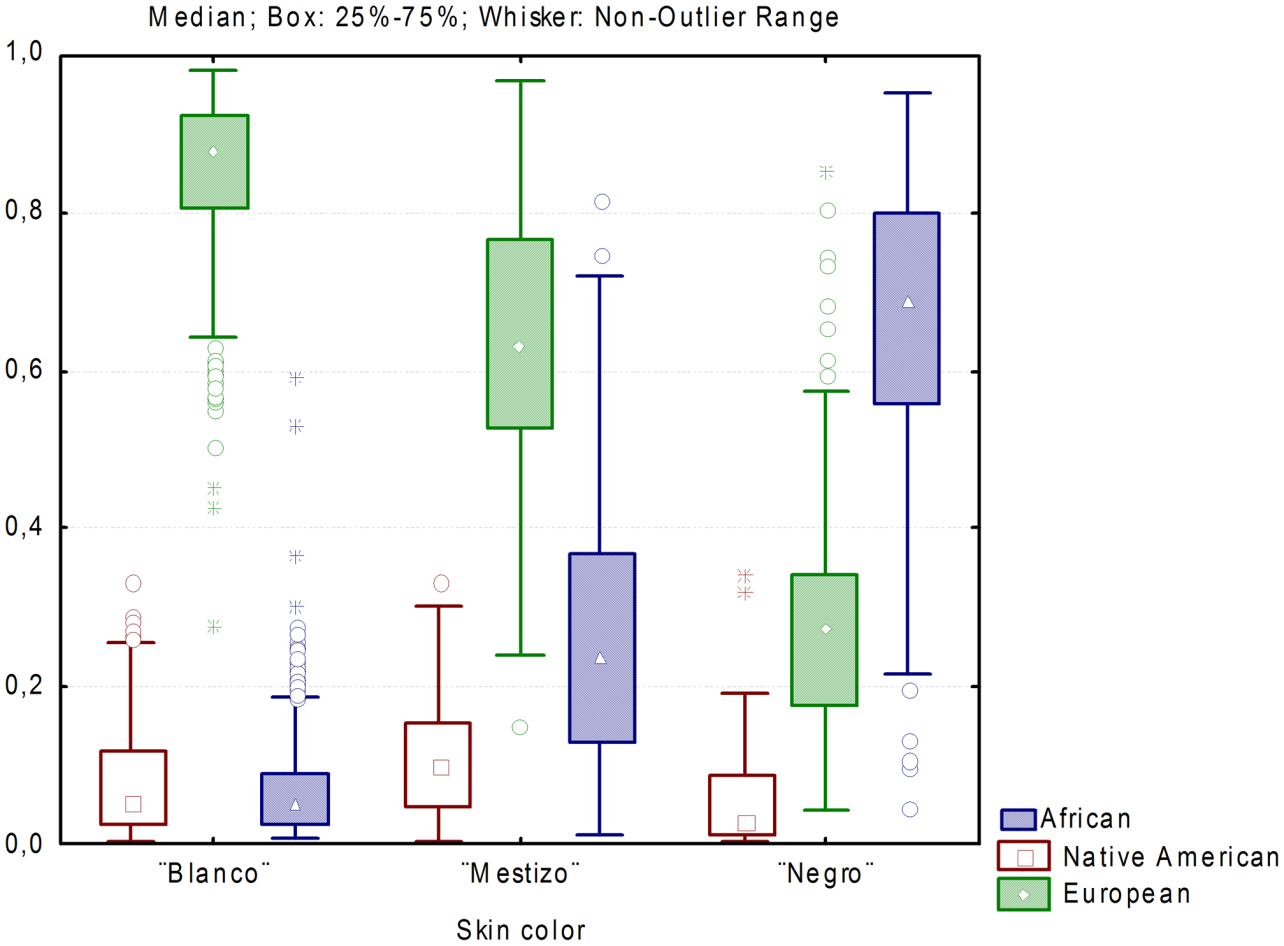
Distribution of individual ancestry proportions stratified by census categories.

The average European, African and Native American ancestry in those self-reporting to be “blanco” were 86%, 6.7% and 7.8%, in those self-reporting to be “mestizo” 63.8%, 25.5% and 10.7%, and in those self-reporting to be “negro” 29%, 65.5%, 5.5% (Figure 3). (ANOVA European: *F* = 1048.04, *P*<0.001; African: *F* = 1138.97, *P*<0.001; Native American: *F* = 34.19, *P*<0.001).

The levels of pigmentation show a strong correlation with the estimates of individual ancestry proportions obtained with the panel of AIMs. African ancestry was positively correlated with the melanin index (Spearman's *rho* = 0.632, *P*<0.001), and European ancestry was inversely correlated with melanin index (*rho* = −0.659, *P*<0.001). No significant correlation was observed between Native American ancestry and melanin index (*rho* = 0.0547, *P* = 0.0809).

**Figure 4:**
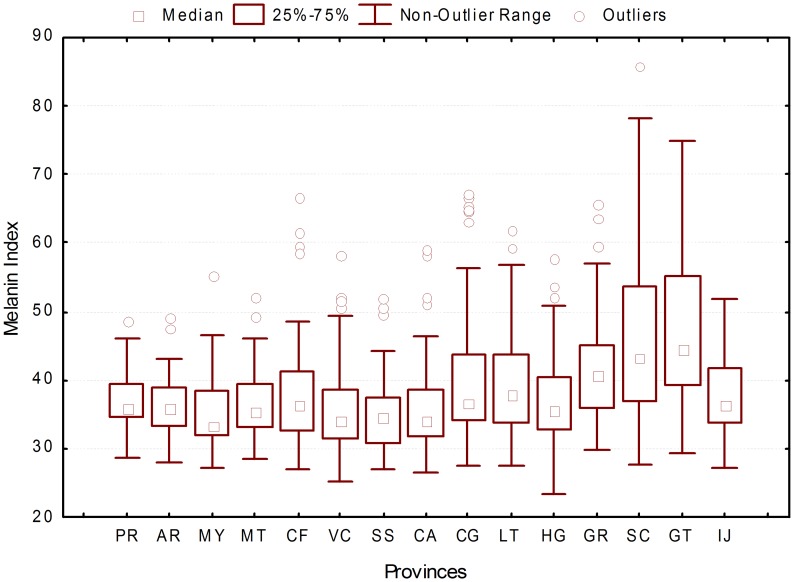
Distribution of melanin index by province.

The analysis of melanin index distribution by province revealed that the samples from Guantánamo (GT) and Santiago de Cuba (SC) have significantly higher melanin index values (GT average M = 47.51, SC average M = 46.77) (Figure 4).

### Exploring potential reasons for geographic patterns in admixture proportions

The data show clear geographic trends in admixture proportions in Cuba. For example, the average African ancestry in the provinces of Guantánamo and Santiago de Cuba is higher than in the other provinces. In principle, this could be explained by two different scenarios, which are not mutually exclusive: (i) African admixture proportions are higher in Guantánamo and Santiago de Cuba because these provinces have higher proportions of individuals self-reporting to be “negro” or “mestizo”, who on average have higher African contributions than individuals reporting to be “blanco”, or (ii). There are no differences in the proportion of individuals self-reporting to be “blanco”, “mestizo” or “negro” between Guantánamo and Santiago de Cuba and the other provinces, but the average African admixture contributions in at least some of the census categories are higher in Guantánamo and Santiago de Cuba than in the other provinces. In order to evaluate these two scenarios, we explored the relationships between African admixture proportions and the proportion of individuals in each province reporting to be “negro”, “mestizo”, or “blanco”. We observed a strong positive relationship between average African ancestry in each province and the proportion of individuals reporting to be “negro” or “mestizo” (r^2^ = 0.69, *P* = 7×10^−5^, and r^2^ = 0.63, *P* = 0.63, *P* = 2×10^−4^, see also Figure S1). Therefore, the higher African admixture proportions in Guantánamo and Santiago de Cuba are due, to a considerable extent, to the higher proportions of self-reported “negro” and “mestizo” in these provinces. We also observed a positive relationship between the proportion of individuals reporting to be “mestizo” and Native American ancestry across provinces, although this relationship is not as strong as that observed for African ancestry (r^2^ = 0.43, *P* = 6×10^−3^). In addition to the relationship of ancestry and census proportions by province, we also explored to which extent there are differences in admixture proportions within each census category (“blanco”, “mestizo” and “negro”) between provinces (Figure S2). The presence of differences in ancestry proportions within each census category would indicate that provincial differences in ancestry proportions are not only due to differences in the relative proportions of individuals from each census category. We observed some differences in ancestry proportions within census categories. For example, within individuals self-reporting to be “blanco”, the average African admixture proportions are significantly higher in Guantánamo, Santiago de Cuba and Granma than in many other provinces, and within individuals self-reporting to be “negro”, the average African admixture proportions are significantly lower in Las Tunas, Holguín and Granma than in Guantánamo, Santiago de Cuba, Camagüey and La Habana (data not shown).

### Admixture proportions in rural and urban areas

We explored if there are differences in ancestry proportions estimated with AIMs between rural and urban areas. For the total sample, we observed that the African ancestry proportions were significantly higher in urban than rural areas (*P* = 0.003), and conversely, the Native American ancestry proportions were significantly higher in rural than urban areas (*P* = 2×10^−6^) (Figure S3). A plot showing ancestry proportions in rural and urban areas by province is depicted in Figure S4. The results of a two-way ANOVA and *post-hoc* tests indicate that the difference in African ancestry proportions between urban and rural areas is primarily driven by the higher African ancestry in individuals reporting to be “negro” living in urban areas vs. those living in rural areas. In contrast, the average Native American contribution in individuals self-reporting to be “negro” living in rural areas is higher than in those living in urban areas, and this is the main factor explaining the higher Native American ancestry in rural vs. urban areas. No significant differences between rural and urban areas were observed for African or Native American ancestry for individuals reporting to be “blanco” or “mestizo”.

### mtDNA and Y chromosome analyses

**Figure 5:**
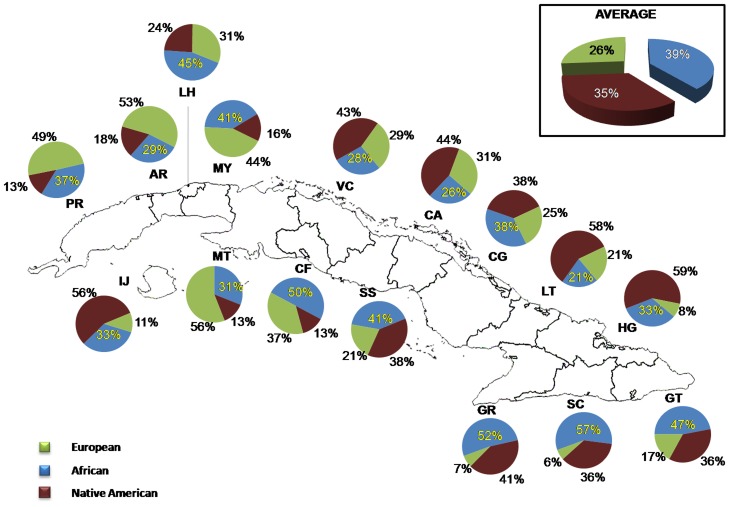
Distribution of ancestral contributions in the total sample and stratified by province as inferred from mtDNA markers.

A total of 943 mtDNA haplotypes could be allocated to a specific branch of the mtDNA phylogeny resolved by the mtSNPs genotyped in the present study (see the mtDNA phylogeny of Figure S5). A detailed list of the haplogroup assignations based on the 18 markers genotyped in this study is presented in Table S5. The analysis of mtSNPs indicates that 34.5% of the mtDNA haplotypes have Native American ancestry, 38.8% African ancestry, and 26.7% Eurasian ancestry (Figure 5). The highest maternal Eurasian proportions were found in the provinces of Matanzas (58%), Artemisa (53%), and Pinar del Rio (49%) and the lowest in Santiago de Cuba (6%), Granma (7%) and Holguín (7.5%). The highest maternal African proportions were observed in the provinces of Santiago (57%) and Granma (52%), and the lowest in Las Tunas (21%) and Camagüey (24%). With respect to the maternal Native American proportions, the highest were found in Holguín (59%) and Las Tunas (58%), and the lowest in Matanzas (13%), Cienfuegos (13%) and Pinar del Río (13%). An analysis of contingency tables using exact tests (Table S6) indicates that many of the Western provinces have significantly higher Eurasian proportions than some of the Eastern provinces, in particular Holguín, Granma and Santiago de Cuba. These tests also show that the province of Santiago de Cuba has significantly higher African proportions than other Cuban provinces, and that the provinces of Holguín, Las Tunas and to some extent, Granma, have significantly higher Native American proportions than most of the Western provinces.

**Figure 6:**
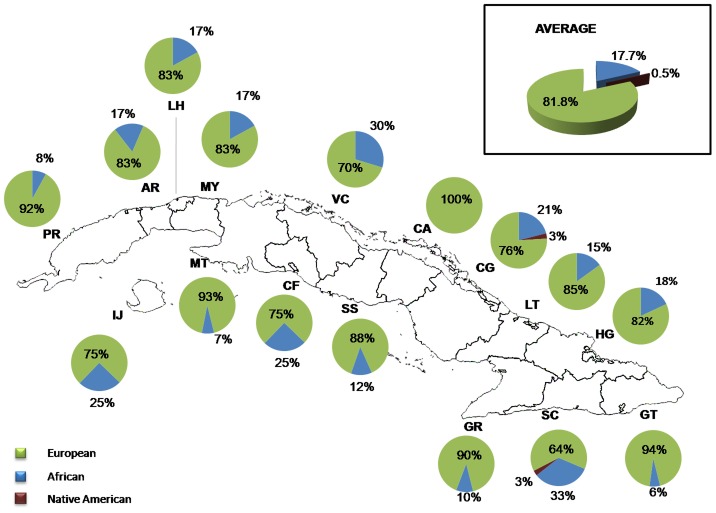
Distribution of ancestral contributions in the total sample and stratified by province as inferred from Y-chromosome markers.

Y-chromosome SNPs could be genotyped in 384 males and haplotypes were classified into haplogroups following the phylogeny of Figure S6. A detailed list of the haplogroup assignations based on the 12 Y-SNPs genotyped in this study is presented in Table S7. Most of the haplotypes are of Eurasian ancestry (81.8%), while 17.7% have African ancestry and only two haplotypes are of Native American ancestry (0.5%) (Figure 6). The Native American haplotypes belong to two individuals, one from the province of Camagüey and the other from Santiago de Cuba. Regarding Eurasian and African ancestry, the highest Eurasian paternal contributions were found in Matanzas, and Pinar del Río, and the highest African paternal contributions correspond to the province of Santiago de Cuba. Although the size of the Y-chromosome sample was substantially smaller than the mtDNA sample, the contingency table analysis (Table S8) identified significant differences in paternal Eurasian contributions between Matanzas and Villa Clara, Cienfuegos and Santiago, and also between Pinar del Río and Guantánamo and Santiago. The province of Santiago showed a significantly higher African paternal contribution than Pinar del Río, Matanzas and Guantánamo.

### Association of genetic markers with melanin levels

Sixteen genetic markers located within or nearby genes that previously have demonstrated association with skin pigmentation (*APBA2* –linked to *OCA2*–, *ASIP*, *BNC2*, *GATA3*, *GRM5* –linked to *TYR*–, *HERC2*–linked to *OCA2*–, *IRF4*, *KITLG*, *MC1R*, *OCA2*, *SLC24A5*, *SLC45A2* –also known as *MATP*–, *TYR*, *TYRP1* and *UGT1A1*) [21]–[37] were analyzed for association with melanin levels measured quantitatively. The program ADMIXMAP was used to run a linear regression analysis conditioning on individual ancestry. Of the 16 markers analyzed, four were significantly associated with melanin index after Bonferroni correction (*P*<0.0031): rs1426654 located on the *SLC24A5* gene (*P* = 1.2×10^−25^), rs16891982 and rs35395 located on the *SLC45A2* (*MATP*) gene (*P* = 1.7×10^−20^ and *P* = 2.8×10^−11^, respectively), and rs12913832 located on the *HERC2* gene, linked to *OCA2* (*P* = 0.0018). In order to evaluate if there was evidence of residual stratification unaccounted for in the analysis based on the three-parental model, we used the *P*-values obtained for 86 AIMs located more than 5 cM apart from the 16 pigmentation markers to estimate the lambda inflation factor. We observed evidence of residual stratification (lambda = 1.38). Therefore, we implemented genome control (GC) [38] methods to correct for type I error inflation. After GC-correction, *SLC24A5* rs1426654 (*P* = 5.1×10^−19^), *SLC45A2* rs16891982 (*P* = 2.9×10^−15^) and *SLC45A2* rs35395 (*P* = 1.5×10^−8^) remained significant after Bonferroni correction. However, the *P*-value for *HERC2* rs12913832 (*P* = 0.0078) slightly exceeded the Bonferroni-corrected threshold. Table 2 reports the GC-corrected *P*-values for all the pigmentation markers. Assuming an additive model, we estimated that each copy of rs1426654 allele A and rs16891982 allele G decrease the melanin index by 5.04 and 3.40 units, respectively. The *HERC2* SNP rs12913832 has a substantially smaller effect, with each copy of the G allele, which has been associated with blue iris color in previous studies [25]–[27], decreasing melanin index by approximately 1.11 units. Finally, we repeated the analysis including the genotypes of rs1426654, rs16891982 and rs12913832 as covariates. This analysis showed that the *P*-value observed for rs35395 at the *SLC45A2* locus was no longer significant, indicating that the significant result for this marker is primarily due to its linkage with rs16891982, which is located approximately 3 kb apart from rs35395 on chromosome 5. None of the other 12 SNPs surveyed had significant effects on melanin levels after conditioning for the rs1426654, rs16891982 and rs12913832 polymorphisms.

**Table 2:** Association of genetic markers within or nearby skin pigmentation genes with melanin levels.

Gene	Locus	*P*-value
*APBA2 (linked to OCA2)*	rs4424881	0.0692
*ASIP*	rs6058017	0.6364
*BNC2*	rs10756819	0.3063
*GATA3*	rs376397	0.7725
*GRM5 (linked to TYR)*	rs10831496	0.3175
*HERC2(linked to OCA2)*	rs12913832	0.0078
*IRF4*	rs12203592	0.1229
*KITLG*	rs642742	0.8327
*MC1R*	rs1805007	0.0896
*OCA2*	rs7495174	0.3069
*SLC24A5*	rs1426654*	**5.1**×**10^−19^**
*SLC45A2/MATP*	rs16891982*	**2.9**×**10^−15^**
*SLC45A2/MATP*	rs35395*	**1.5**×**10^−8^**
*TYR*	rs1042602	0.3313
*TYRP1*	rs2733831	0.8296
*UGT1A1*	rs6742078	0.9097

*Significant level after Bonferroni correction: *P*<0.0031.

The program ADMIXMAP was used to run a linear regression analysis conditioning on individual ancestry. The *P*-values for 86 AIMs unlinked to the pigmentation markers were used to estimate the lambda inflation factor. We report the *P*-values after Genome Control (GC) correction.

## Discussion

Here we report an analysis of the admixture proportions in a large sample from Cuba using a combination of highly informative AIMs, mtSNPs and Y-SNPs. One of the major strengths of this study is the careful selection of the sample, which represents all the provinces of Cuba. The sample comprises individuals from more than 81% of the Cuban municipalities, and the proportions according to province, age group, and rural/urban population are very similar to the proportions reported in the Cuban 2002 census [39]. The distributions of gender and census categories (“blanco”, “mestizo” and “negro”) are slightly different from the reported 2002 census proportions. The proportion of females in the sample (58%) is higher than that reported in the census (50%). This is related to the fact that when the households were visited, relatively more women were the only household members present during the visit. With respect to the census categories, the sample included relatively more individuals classified as “mestizos” and less individuals classified as “blancos” than in the 2002 census (mestizos: 33% vs. 25%, blancos: 55% vs 65%), and the proportion of individuals classified as “negro” was overly similar in the sample and 2002 census (12% vs. 10%). There were also slight differences in the way that the census categories were obtained: In the 2002 census, the “color” categories were classified by the census collectors, and when an individual was not present in the household, census categories were reported by family members. In the present sample, the census categories were obtained in two ways: self-reported and reported by a trained researcher and we observed a very high concordance between the two classifications.

The use of autosomal and maternally and paternally inherited polymorphisms allowed us to carry out a detailed analysis of admixture in Cuba, and the analysis by provinces identified very clear and consistent patterns. Using autosomal AIMs, we observed that the average European, African and Native American proportions in the sample were 72% (SD: ±22,61), 20% (SD: ±22,66) and 8% (SD: ±6,86), respectively. However, the amount of European ancestry tends to be higher in the Western provinces of Cuba than in the Eastern provinces. In contrast, the highest African proportions are observed in the eastern provinces of Santiago de Cuba and Guantánamo and the highest Native American contributions in the Eastern provinces of Las Tunas, Granma and Holguín. Importantly, the results based on analyses of the mtDNA and Y-chromosome SNPs are fully consistent with this picture. The highest Eurasian proportions observed for both the mtDNA and the Y chromosome are found in the Western provinces, particularly Matanzas and Pinar del Río, and the highest African contributions are present in Santiago de Cuba. Regarding the Native American contribution, the mtDNA analysis also indicates that the highest Native American proportions are present in the provinces of Holguín and Las Tunas. We only observed two Native American Q-M3 haplogroups in the male sample, corresponding to individuals from the provinces of Camagüey (in the Central region of the island) and Santiago de Cuba (in the East).

Our analyses indicate that the geographic trends observed in ancestry proportions are due, at least to some extent, to differences in the relative proportions of individuals reporting to be “blanco”, “mestizo” or “negro” across provinces. The provinces of Guantánamo and Santiago de Cuba, which show the highest average African ancestry and melanin index levels, also have the highest proportion of individuals self-reporting to be “mestizo” and “negro”. However, this does not seem to be the only reason behind these differences. We also observe that there are some differences in average admixture proportions within each census group between provinces. For example, the average African ancestry of individuals self-reporting to be “blanco” tends to be higher in Guantánamo, Santiago de Cuba and Granma than in other provinces. In general, our study highlights the subjectivity involved in the categories “blanco”, “mestizo” and “negro”. Although there are significant differences in melanin index between the three categories, there is some overlap in melanin values between these groups. This means that two individuals with the same melanin index values may report different census categories (e.g. “blanco” or “mestizo”). In addition to the analyses by province, we also evaluated the distribution of ancestry proportions in urban vs. rural areas. We observed that the African ancestry proportions were significantly higher in urban than rural areas, and conversely, the Native American ancestry proportions were significantly higher in rural than urban areas.

The geographic patterns observed in the distribution of admixture proportions are in agreement with historical and archaeological data. It is known that at the arrival of the Spaniards to Cuba, the Taino primarily inhabited the eastern regions of Cuba. Estimates of the population distribution in the year 1510 indicate that more than 50% of the indigenous Cuban population lived in the eastern region (from Las Tunas to Guantánamo), less than 40% lived in Camagüey and Las Villas (both in the central region of Cuba), and less than 10% inhabited the western region of the island. Within the eastern region, Holguín was the most populated area, followed by the region of Bayamo (currently the province of Granma) [40]. The results of our study, which reveal that the province of Holguín has some of the highest autosomal and mtDNA Native American proportions in Cuba, are therefore in agreement with the historical sources described above and the high concentration of Taino archaeological sites in this area [41]. Historical reports indicate that the indigenous population collapsed from more than 100,000 at the arrival of the Europeans to 2,000–3,000 in 1556, primarily due to the harsh conditions of forced labor, the disruption of the agricultural system and the epidemic diseases brought by the Europeans [39], [42]. In the early stages of colonization there was immigration from Europe, primarily from the Iberian Peninsula and the Canary Islands, and enslaved Africans were also brought to the island. Initially, the number of enslaved Africans was small, but increased substantially in the final period of the 18^th^ century [40]. Although the eastern region was, at the arrival of the Europeans, the most populated region of the island, this was the region that took the longest to repopulate after the demographic collapse that occurred during the first stages of colonization. The western region had an important number of enslaved Africans working in the sugar plantations, but this was also the region that received most of the immigrants from the Iberian Peninsula [40]. In contrast to the western region, where most of the enslaved Africans came directly from Africa, in the eastern region many of the individuals of African ancestry came from Jamaica and Haiti, and were forced to work in coffee and sugar plantations. Historical sources indicate that in 1830 there were more than 50,000 enslaved Africans in Santiago de Cuba and Guantánamo, the regions where we have identified the highest African contributions [43], [44].

The comparison of the relative autosomal, paternal and maternal admixture proportions clearly show that the process of admixture in Cuba has been sex-biased, with a relatively higher European contribution observed for the paternal lineages, and a higher African and Native American contribution in the maternal lineages. This sex-biased contribution is particularly evident for the Native American ancestry. We estimated the average maternal Native American proportion to be 34.5% in the sample, in sharp contrast to the autosomal (8%) and paternal (0.5%) proportions. The African maternal, autosomal and paternal proportions were estimated to be 39%, 20% and 18%, respectively. Overall, our results are very similar to those obtained in an independent study that analyzed mtDNA and Y-chromosome variation in a Cuban sample comprising 245 individuals [9]. In this study, the authors reported that 45% of the mtDNA lineages were of African ancestry and 33% of Native American ancestry. In contrast, only 20% of the Y-chromosome lineages were of African ancestry, and the authors did not find any Y-chromosome Native American lineages. Thus, the genetic data confirms historical information indicating that most of the European migrants to Cuba were males, and that the process of mixing primarily took place between European males and Native American females, during the first stages of colonization, and African females during the slave trade period [5], [9], [12], [13].

We explored the relationship between admixture estimates based on genetic markers, melanin levels measured with a reflectometer, and self-reported census categories (“blanco”, “mestizo” and “negro”). We observed strong relationships between admixture proportions and melanin levels, admixture proportions and census categories, and melanin levels and census categories (see Results section). Overall, these analyses show that there is very substantial population stratification in the current Cuban population, both across and also within self-reported census categories, emphasizing the need to control for the effects of population stratification in association studies in this population. A clear example of the consequences of stratification can be seen in an analysis of the results of a linear regression model without conditioning for individual ancestry proportions, based on 86 AIMs that are located more than 5 cM apart from any of the pigmentation markers analyzed in this study. In such analysis, 64 of the 86 AIMs (74.4%) surpass the Bonferroni-corrected significance threshold (*P* = 5.8×10^−4^). In contrast, none of the AIMs surpass this threshold when the analysis is carried out conditioning on individual ancestry. This implies that in case-control studies in which there are differences in ancestry proportions between the case and control group, or association analysis of quantitative traits that have different distributions in the parental populations, such as pigmentation, there would be a dramatic inflation in the number of false positives. We observed that even after conditioning for individual ancestry there was evidence of residual stratification in the Cuban sample, although of relatively small magnitude (lambda 1.38). Consequently, the *P*-values observed for the pigmentation markers were corrected using Genome Control (GC) strategies.

Finally, we also evaluated the association of 16 SNPs located within or nearby pigmentation genes with melanin levels (e.g. melanin index). These polymorphisms have been associated with pigmentary phenotypes in previous studies [21]–[37]. Our analysis confirms previously reported associations of rs1426654, located within the *SLC24A5* gene (*P* = 5.1×10^−19^) and rs16891982, located within the *SLC45A2* (*MATP*) gene (*P* = 2.9×10^−15^) with skin pigmentation. These two markers have the strongest effects on melanin levels described in human populations, and in our study we estimated that each copy of rs1426654 allele A and rs16891982 allele G decrease the melanin index by 5.04 and 3.40 units, respectively. The marker rs12913832, which is located within the *HERC2* gene and is known to affect the transcription of the *OCA2* gene, showed a significant effect in the initial ADMIXMAP association tests (*P* = 0.0018), but it did not surpass the Bonferroni-corrected threshold (P<0.0033) after GC-correction (*P* = 0.0077). This marker is strongly associated with blue eye color in European populations [25]–[27], but it has also been associated with skin pigmentation, tanning response and hair color in previous studies [28]–[30].

One of the limitations of this study is the relatively small number of genetic markers used to characterize admixture proportions. We employed 128 autosomal AIMs to identify ancestral contributions, and this panel should be sufficient to obtain precise admixture estimates for the overall sample and the provinces. However, the precision of the individual admixture estimates is not comparable with the precision that can be achieved with dense microarrays. Unfortunately, we do not have genome-wide data to evaluate the precision of our individual admixture estimates. An indirect estimate can be obtained through comparison with a sample from Puerto Rico [45], which has very similar average admixture proportions as our sample (average European ancestry: 67%, average African ancestry: 21% and average Native American ancestry: 12%), and was characterized with a genome-wide panel, in addition to a panel of AIMs that greatly overlaps with the panel used in this study (105 AIMs common in both studies). Galanter et al. [45] described the correlation of the individual admixture estimates based on 84 AIMs and 194 AIMs with the estimates based on genome-wide data. For 84 AIMs, the r^2^ values for European, African and Native American ancestry were 0.72, 0.72 and 0.27, respectively. For 194 AIMs, the r^2^ values for European, African and Native American ancestry were 0.85, 0.89 and 0.43. The lower r^2^ values observed for Native American ancestry are primarily due to the low overall Native American proportions observed in the Puerto Rican sample (similarly, in a sample from Mexico, substantially lower r^2^ values were observed for African ancestry than for European and Native American ancestry, due to the low overall African proportions observed in this sample). Therefore, based on the Puerto Rican data, we can infer that our panel of AIMs should provide reasonable estimates of European and African contributions at the individual level (r^2^ with estimates based on genome-wide data close to 0.8), although the precision for the Native American ancestral component is probably substantially lower (r^2^<0.4). These r^2^ values give an indication of the average precision of the individual ancestry in the full sample, but there will be some variation in the level of concordance between the genome-wide and the AIMs estimates for each individual. With respect to the estimates of maternal and paternal contributions, the number of markers characterized in the sample is enough to obtain adequate estimates of ancestral contributions at the continental level, but given the relatively low number of diagnostic sites the resolution of the haplogroups is phylogenetically low; therefore a much more extensive analysis would be necessary in order to obtain a more precise picture of the mtDNA and Y-specific lineages present in the Cuban population. We hope that future studies of this sample using microarray platforms, and a much more extensive characterization of the mtDNA (e.g. sequencing the whole molecule) and Y-chromosome will make it possible to obtain a more complete perspective of the complex history of the Cuban population, expanding the current level of resolution from the continental to the intra-continental level (e.g. relative ancestral contributions of populations within continents). A recent paper by Moreno-Estrada et al. [44] has shown the increased resolution that can be obtained with dense microarray data.

### Conclusion

By genotyping a panel of autosomal AIMs in combination with mtDNA and Y-chromosome markers in a large sample representative of all Cuban provinces, we were able to identify very clear patterns in the distribution of admixture proportions throughout Cuba. The analysis using AIMs indicated that the average European, African and Native American contributions were 72%, 20% and 8%, respectively. However, the African and Native American contributions were relatively higher, and the European contributions lower, in the Eastern provinces than in the Western provinces. In particular, the Southeastern provinces, such as Santiago de Cuba and Guantánamo, showed the highest African proportions, and the highest Native American proportions were found in the Eastern provinces of Granma, Holguín and Las Tunas. Similar geographic patterns were observed in the analyses of the uniparental markers. Additionally, by comparing the autosomal and uniparental admixture proportions, we identified a clear sex-biased pattern in the process of gene flow, with a substantially higher European contribution from the paternal side than the maternal side, and conversely higher Native American and African contributions from the maternal side than the paternal side. The geographic patterns observed for the admixture proportions are consistent with historical and archaeological evidence. The identification of sex-biased gene flow is also in agreement with historical information indicating that most of the European immigrants throughout Cuban history were male and that the process of admixture took place primarily between European males and Native American and African females. Finally, we observed that SNPs located in the genes *SLC24A5*and *SLC45A2* are significantly associated with skin pigmentation in the sample, in accordance with what has been reported in other admixed populations.

## Materials and Methods

### Ethics statement

The study was approved by the Research Ethics Committee of the National Centre of Medical Genetics of Cuba. Each individual in this study gave written informed consent prior to the interview, physical examination and blood sample collection.

### Sample

The final sample comprised 1,019 individuals representing all the provinces of Cuba. The selection of the individuals was made in collaboration with the National Statistics Office from Cuba. The individuals were selected based on the demographic characteristics of the Cuban population in terms of population density, age, gender and census category (“Blanco”, “Mestizo”, “Negro”). Individuals were recruited from 1,229 households, located in 137 of the 168 Cuban municipalities. Selection of individuals from each household was based on the Kish grid, in order to ensure that all the members of the household had the same probability of being selected for the study. The final sample represents quite well the current distribution of the Cuban population in terms of sex, age, census category (“Blanco”, “Mestizo”, and “Negro”), provincial population density and rural/urban residence. A detailed comparison of the relative proportions of each category in the study sample and the Cuban census is provided as supplementary information (see Table S1). Researchers visited 1,182 of the 1,229 selected households and 1,031 individuals volunteered to participate in the study. Due to problems with DNA quality, 12 samples were excluded from the final analyses.

Information about individual, parent and grandparents place of birth, demographics, education level, physical health, mental disorders, non-communicable disease risk factors and anthropometry was collected *via* questionnaire and physical examination. Information about census category was obtained in two ways: self-reported by the participants and independently classified by one trained researcher (EFS) for all the individuals included in the study.The concordance between the two classifications was evaluated using Cohen's *kappa* coefficient [17], and also the Ciccheti-Allison [18] and Fleiss-Cohen [19] weighted *kappa* coefficients.

### Measurement of skin pigmentation

Melanin content of the skin was measured with a narrow band reflectometer (DSM II ColorMeter, Cortex Technologies, Hadsund, Denmark) [20]. This instrument provides quantitative estimates of melanin levels (e.g. melanin index). The measurements were taken at the medial side of the upper inner arm, an area of the body not exposed to the sun (constitutive pigmentation), and also at the dorsum of the hand, an area with substantial exposure to the sun (facultative pigmentation).

### Genetic markers

#### a) Autosomal markers

In order to estimate genomic ancestry, 128 AIMs were genotyped using the SequenomMassARRAY Genotyping platform (Sequenom, San Diego, CA) (See Table S2 for a full list of the AIMs genotyped in the study). This panel of AIMs includes some of the most informative markers described in a recent study published by Galanter et al. [45]. Additionally, 16 SNPs located in 15 genes that have been associated with pigmentary phenotypes in previous studies [21]–[37] were genotyped using the same platform. (See Table S3 for information about the SNPs and the pigmentation genes).

#### b) Mitochondrial DNA markers

A total of 18 mtDNA SNPs (mtSNPs) were genotyped. MtSNPs were selected from a wider mtSNP panel published by Álvarez-Iglesias et al. [46], [47], with minor changes on primer designs (details are provided in Table S4). Haplogrouping (*sensu* [48]) was carried out using as reference the worldwide mtDNA phylogeny provided by PhyloTree Build 15 [49]. The revised Cambridge Reference Sequence (rCRS) [50] was taken as reference instead of the Reconstructed Sapiens Reference Sequence or RSRS [51]. Profiles were checked for potential genotyping errors following the procedures described by Salas et al. [52].

#### c) Y-chromosome markers

We analyzed 12 Y-SNPs, namely, M22, 92R7, SRY1532, M70, M173, Tat, M213, M9, M269, M173, M242, M3. These SNPs were selected from a wider panel of SNPs described in Brión et al. [53] and Blanco-Verea et al. [54]. All markers were genotyped in one multiplex reaction following conditions described in Blanco-Verea et al. [54]. Haplotypes were allocated into haplogroups following the nomenclature of the Y-Chromosome Consortium (http://www.isogg.org/wiki/Y_Chromosome_Consortium).

### Analysis of admixture proportions and association of genetic markers with quantitative measures of skin pigmentation

Average admixture proportions, the sum of intensities parameter (equivalent to the average number of generations since the admixture event) and the individual ancestry proportions were estimated using the software ADMIXMAP v3.8 for Windows. This is a general purpose program for modeling population admixture with genotype and phenotype data, based on a combination of Bayesian and classical methods. If information for a quantitative trait (such as skin pigmentation) is provided, ADMIXMAP fits a linear regression model of the trait conditioning upon individual admixture. Covariates such as sex and age can be included in this model. Detailed information about this program can be found in Hoggart et al. [55], [56]. In order to estimate admixture proportions; we used the prior allele frequency model, which requires information about the prior distribution of allele frequencies in each ancestral population. Under this model, the program estimates the allele frequencies from unadmixed and admixed population samples simultaneously, allowing for sampling error. ADMIXMAP implements a diagnostic test for variation of allele frequencies between the unadmixed populations that were sampled to obtain prior parameters and the corresponding ancestry-specific allele frequencies in the admixed sample. The program was run with 20,000 iterations, including 1,000 iterations for burn-in of the Markov chain.

### Statistical analysis

Differences between provinces and between sexes for the melanin index and the ancestral genetic proportions were assessed using one-way ANOVA. The relationship between age and skin pigmentation was assessed by the parametric Pearson correlation test and also the non-parametric Spearman's *rho* test. A two-way ANOVA was conducted in order to evaluate the relationship between melanin index and skin color using sex as a covariate. Finally, potential differences in the distributions of mtDNA and Y-chromosome haplogroups among provinces were evaluated using exact tests. The above described statistical analyses were performed in Statistic 7.0 and SPSS 20.0.

## Supporting Information

Figure S1 Relationships between admixture proportions estimated with AIMs and census categories:”negro”, “mestizo”, “blanco”.
(TIF)

Figure S2 Plot of admixture proportions estimated with AIMs and census category: “negro”,“mestizo” and “blanco”, by province.
(TIF)

Figure S3 Plot of admixture proportions: African, Native American, European, estimated with AIMs in urban and rural areas.
(TIF)

Figure S4 Plot of admixture proportions: African, Native American, European estimated with AIMs in urban/rural areas by province.
(TIF)

Figure S5 mtDNA phylogeny.
(TIF)

Figure S6 Y-chromosome phylogeny.
(TIF)

Table S1 Comparison of demographic characteristics in the study sample and the Cuban census from 2002.
(DOCX)

Table S2 Autosomal AIMs genotyped in the study.
(DOCX)

Table S3 SNPs on pigmentation genes.
(DOCX)

Table S4 Primers designs of the mtSNPs genotyped in the present study.
(XLSX)

Table S5 Haplogroup assignations based on 18 mtDNA markers.
(DOCX)

Table S6 Contingency table analysis for mtDNA.
(DOCX)

Table S7 Haplogroup assignations based on Y-chromosome markers.
(DOCX)

Table S8 Contingency table analysis for Y-chromosome.
(DOCX)
