# Supplementary figures and images for: Cuba: Exploring the History of Admixture and the Genetic Basis of Pigmentation Using Autosomal and Uniparental Markers
Source: PLoS Genet. 2014 Jul 24;10(7):e1004488. doi: 10.1371/journal.pgen.1004488 (PMC4109857; doi:10.1371/journal.pgen.1004488)

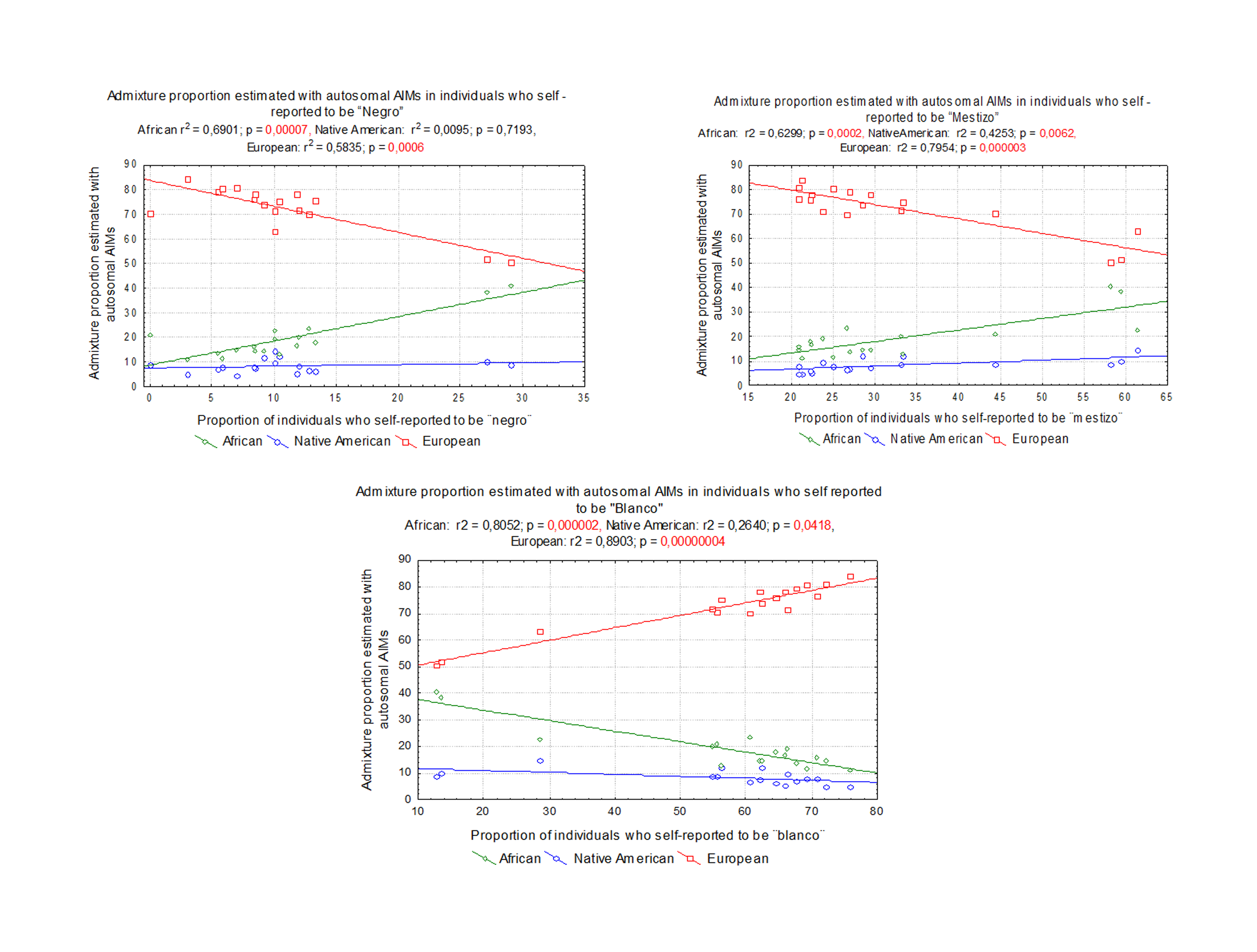

Supplement: Figure S1 — Relationships between admixture proportions estimated with AIMs and census categories:”negro”, “mestizo”, “blanco”. (TIF) [file pgen.1004488.s001.tif]

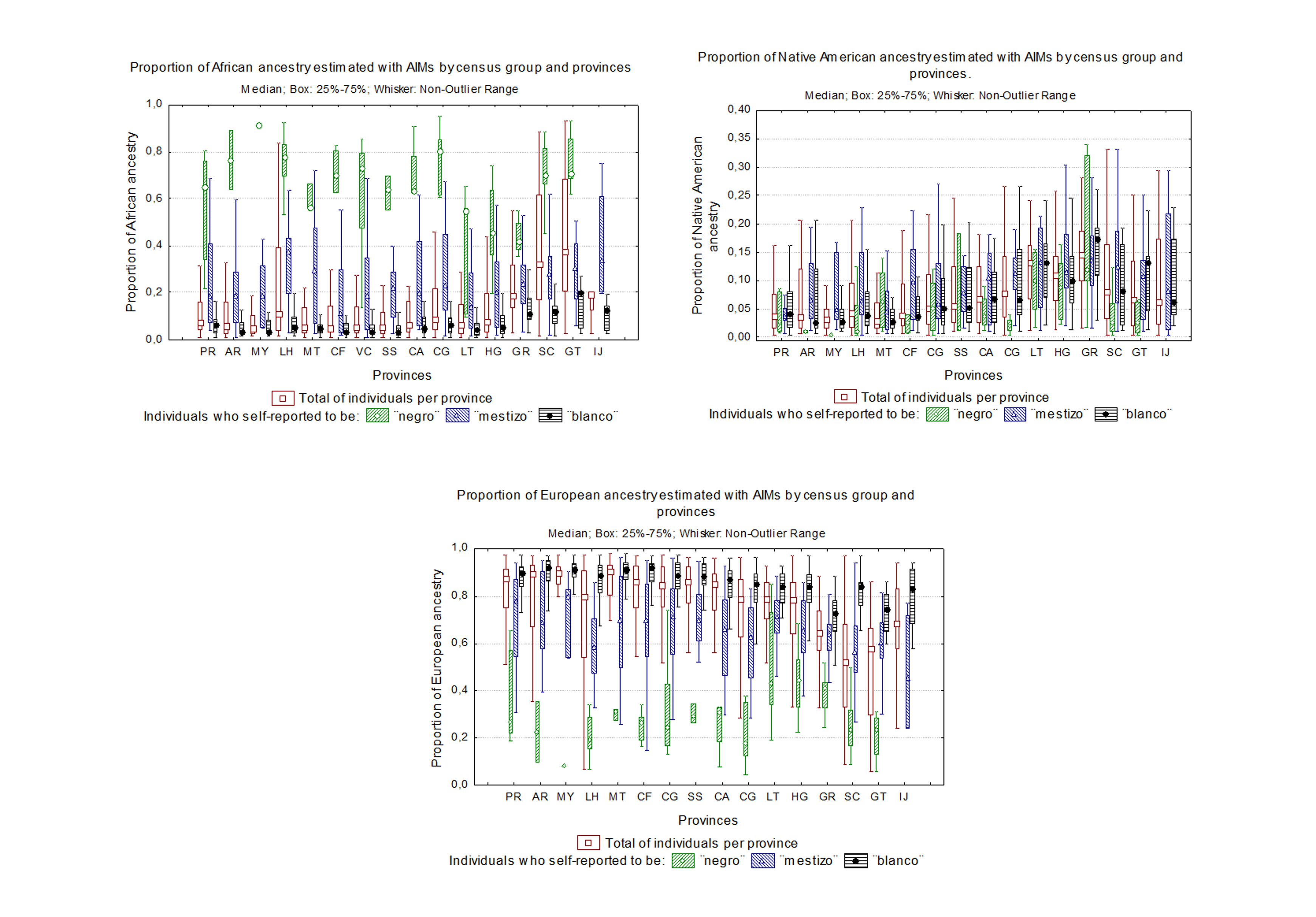

Supplement: Figure S2 — Plot of admixture proportions estimated with AIMs and census category: “negro”,“mestizo” and “blanco”, by province. (TIF) [file pgen.1004488.s002.tif]

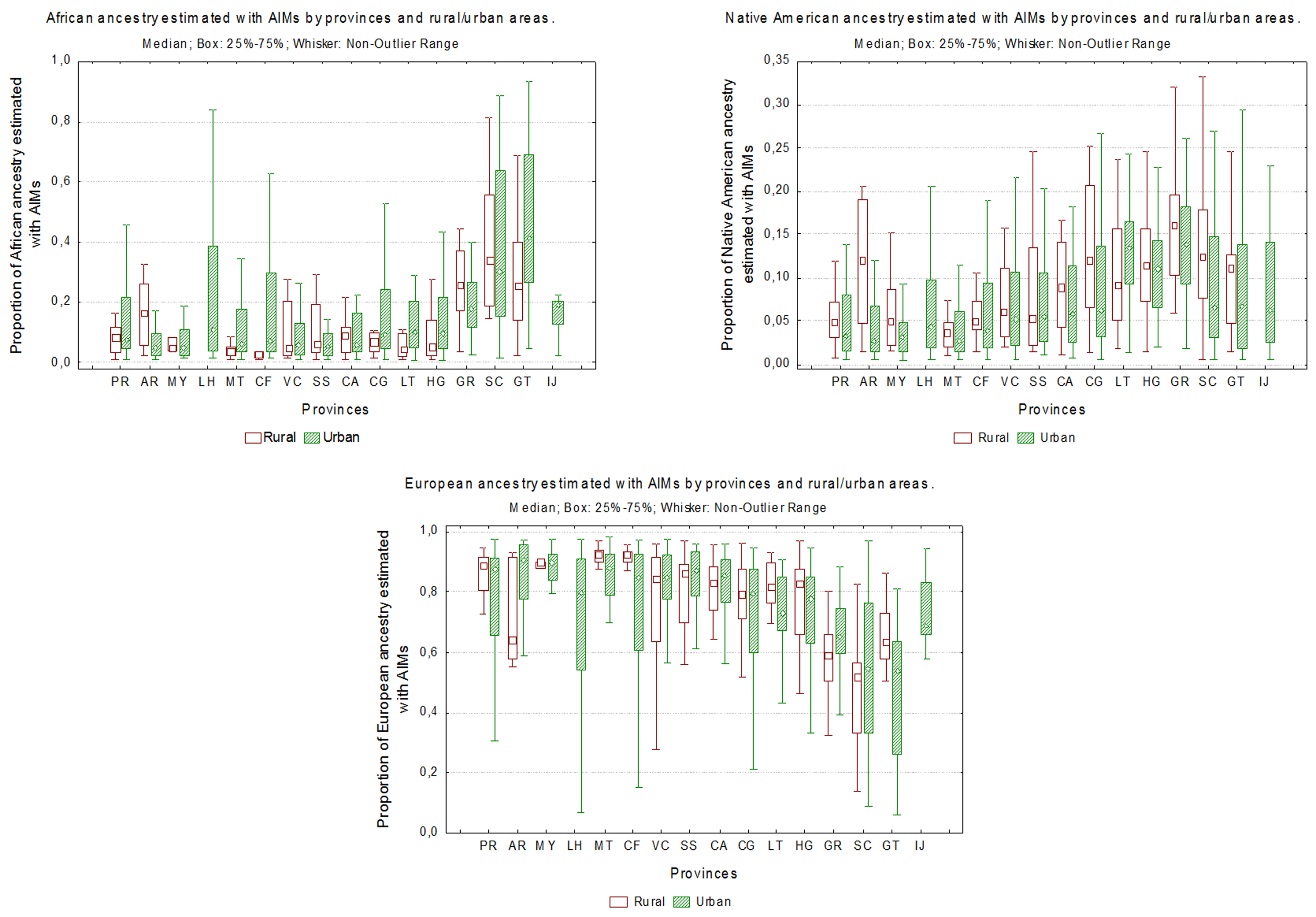

Supplement: Figure S4 — Plot of admixture proportions: African, Native American, European estimated with AIMs in urban/rural areas by province. (TIF) [file pgen.1004488.s004.tif]

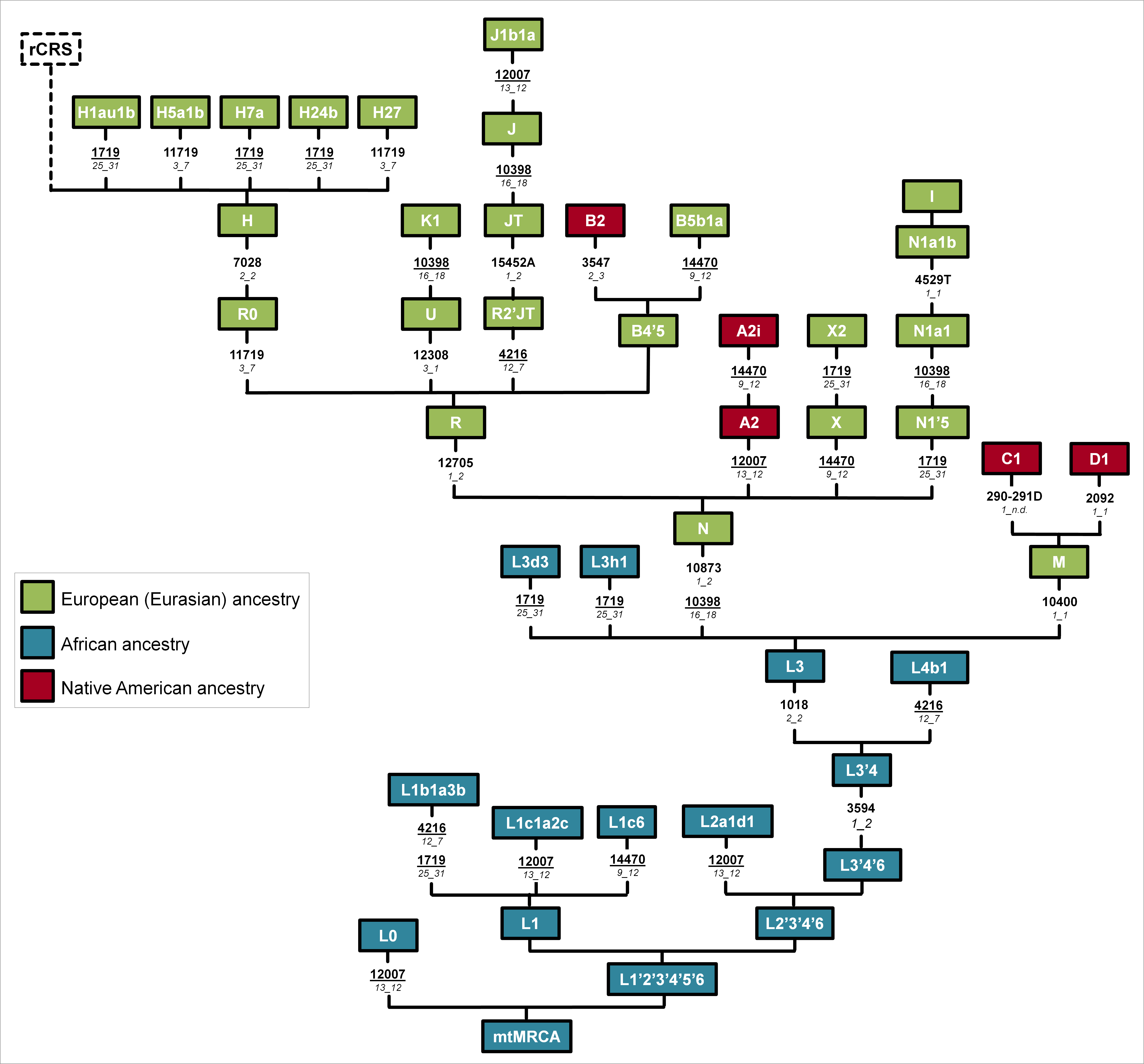

Supplement: Figure S5 — mtDNA phylogeny. (TIF) [file pgen.1004488.s005.tif]

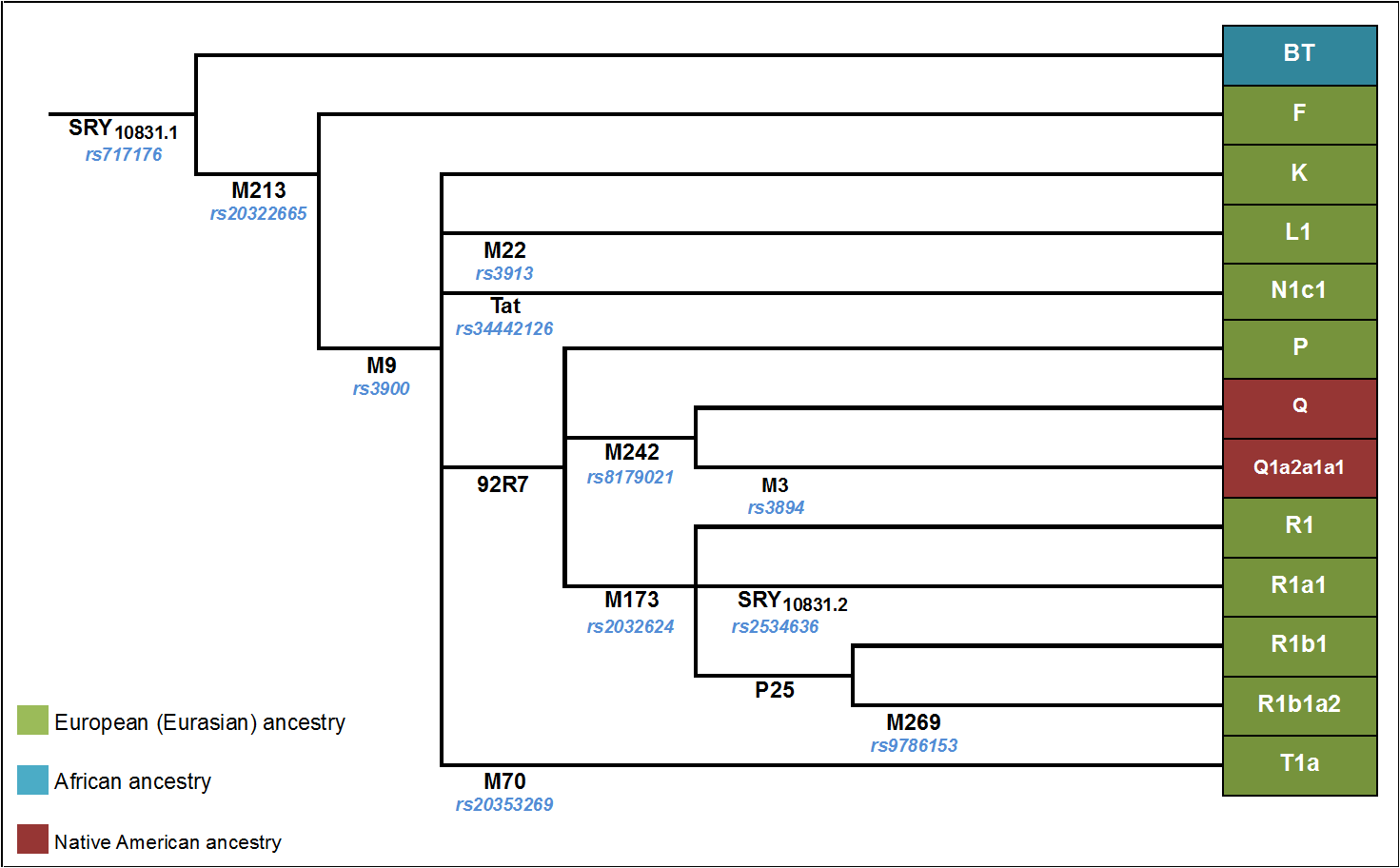

Supplement: Figure S6 — Y-chromosome phylogeny. (TIF) [file pgen.1004488.s006.tif]
