# Supplementary material for: Cuba: Exploring the History of Admixture and the Genetic Basis of Pigmentation Using Autosomal and Uniparental Markers
Source: PLoS Genet. 2014 Jul 24;10(7):e1004488. doi: 10.1371/journal.pgen.1004488 (PMC4109857; doi:10.1371/journal.pgen.1004488)
Supplement: Table S1 — Comparison of demographic characteristics in the study sample and the Cuban census from 2002. (DOCX) [file pgen.1004488.s007.docx]

| **Demographic characteristics** | | **Data from the Cuban Census 2002** | | **Data from the Present Study** | |
| --- | --- | --- | --- | --- | --- |
|  |  | **Absolute Frequency** | **Relative Frequency** | **Absolute Frequency** | **Relative**  **Frequency** |
| **Sex** | Male | 5.597.233 | 50.1 | 429 | 42.0 |
|  | Female | 5.580.510 | 49.9 | 590 | 58.0 |
| **Skin Color** | White or ‘Blancos’ | 7.271.926 | 65.0 | 560 | 55.0 |
|  | Mixed or ‘Mestizos’ | 2.778.923 | 24.9 | 337 | 33.0 |
|  | Black or ‘Negros’ | 1.126.894 | 10.1 | 122 | 12.0 |
| **Place of Residence** | Urban | 8.430.871 | 75.0 | 784 | 77.0 |
|  | Rural | 2.810.290 | 25.0 | 235 | 23.0 |
| **Age of participants** | 18-19 yrs | 313.207 | 0.03 | 17 | 0.02 |
|  | 20- 29 yrs | 1.560.684 | 14.0 | 120 | 11.8 |
|  | 30-39 yrs | 2.198.106 | 19.7 | 156 | 15.3 |
|  | 40-49 yrs | 147.8951 | 13.2 | 253 | 24.8 |
|  | 50-59 yrs | 119.6596 | 10.7 | 186 | 18.1 |
|  | 60-69 yrs | 844.148 | 7.5 | 152 | 15.1 |
|  | 70-79 yrs | 512.803 | 4.6 | 97 | 9.5 |
|  | 80-84 yrs | 149.688 | 1.3 | 24 | 2.3 |
|  | + 85 | 132.623 | 1.2 | 14 | 1.4 |
| **Population by province** | Pinar del Río (PR) | 728.297 | 6.5 | 76 | 7.5 |
|  | Artemisa (AR)* | 749.289 | 6.7 | 70 | 6.9 |
|  | Mayabeque (MY)* |  |  |  |  |
|  | La Habana (LH) | 2.135.498 | 19.0 | 94 | 9.2 |
|  | Matanzas (MT) | 692.536 | 6.2 | 72 | 7.1 |
|  | Cienfuegos (CF) | 407.189 | 3.6 | 45 | 4.4 |
|  | Villa Clara (VC) | 800.335 | 7.1 | 95 | 9.3 |
|  | Sancti Spíritus (SS) | 465.674 | 4.1 | 52 | 5.1 |
|  | Ciego de Ávila (CA) | 424.245 | 3.8 | 48 | 4.7 |
|  | Camagüey (CG) | 780.598 | 6.9 | 80 | 7.9 |
|  | Las Tunas (LT) | 538.062 | 4.8 | 48 | 4.7 |
|  | Holguín (HG) | 1.037.573 | 9.2 | 109 | 10.7 |
|  | Granma (GR) | 836.366 | 7.4 | 70 | 6.9 |
|  | Santiago de Cuba (SC) | 1.047.963 | 9.3 | 96 | 9.4 |
|  | Guantánamo (GT) | 511.116 | 4.5 | 55 | 5.4 |
|  | Isla de la Juventud (IJ) | 86.420 | 0.8 | 9 | 0.9 |

**Table S1.** Comparison of demographic characteristics in the study sample and the Cuban census from 2002.

*At the moment of the design of the study, the provinces of Artemisa (AR) and Mayabeque (MY) were part of the same province, which was later divided in these two new provinces during a recent political reorganization of the Cuban provinces.
