# Supplementary material for: Cuba: Exploring the History of Admixture and the Genetic Basis of Pigmentation Using Autosomal and Uniparental Markers
Source: PLoS Genet. 2014 Jul 24;10(7):e1004488. doi: 10.1371/journal.pgen.1004488 (PMC4109857; doi:10.1371/journal.pgen.1004488)
Supplement: Table S2 — Autosomal AIMs genotyped in the study. (DOCX) [file pgen.1004488.s008.docx]

**Table S2.** Autosomal AIMs genotyped in the study

| **LocusName** | **Chromosome** | **Position in bp** | **Position in cM** |
| --- | --- | --- | --- |
| rs6684063 | chr01 | 30368433 | 59.802.648 |
| rs12021830 | chr01 | 119274424 | 151.900.381 |
| rs2814778 | chr01 | 155987756 | 164.822.103 |
| rs2806424 | chr01 | 159423117 | 171.312.282 |
| rs1780349 | chr01 | 161340963 | 173.884.583 |
| rs6003 | chr01 | 193762678 | 211.973.061 |
| rs10779334 | chr01 | 215494799 | 240.715.819 |
| rs1861498 | chr02 | 8026219 | 18.206.342 |
| rs6711746 | chr02 | 29350200 | 51.221.566 |
| rs10175357 | chr02 | 35073323 | 59.090.117 |
| rs305163 | chr02 | 36221313 | 59.629.105 |
| rs3287 | chr02 | 54719308 | 79.313.151 |
| rs7594727 | chr02 | 96911744 | 112.492.888 |
| rs1567803 | chr02 | 100801536 | 114.362.823 |
| rs1196705 | chr02 | 150044730 | 162.396.667 |
| rs298259 | chr02 | 157150333 | 169.016.437 |
| rs1809119 | chr02 | 174351436 | 184.691.477 |
| rs6710083 | chr02 | 192598098 | 197.304.571 |
| rs16851773 | chr02 | 215014315 | 213.942.343 |
| rs6768750 | chr03 | 7215675 | 20.158.570 |
| rs6803290 | chr03 | 59667208 | 81.611.288 |
| rs862500 | chr03 | 64247689 | 90.046.657 |
| rs13097560 | chr03 | 85212019 | 118.346.283 |
| rs7630522 | chr03 | 108635778 | 130.872.850 |
| rs2937673 | chr03 | 118715077 | 140.389.406 |
| rs1586861 | chr03 | 140541194 | 160.195.996 |
| rs7665516 | chr04 | 11310861 | 24.632.912 |
| rs10026397 | chr04 | 41406815 | 67.535.884 |
| rs924780 | chr04 | 149043602 | 162.621.914 |
| rs1403454 | chr04 | 150092573 | 163.491.982 |
| rs7664076 | chr04 | 172763458 | 193.028.691 |
| rs353373 | chr05 | 40261722 | 64.246.557 |
| rs3309 | chr05 | 56128536 | 70.454.438 |
| rs349323 | chr05 | 100804840 | 112.059.206 |
| rs6866970 | chr05 | 100978603 | 112.098.611 |
| rs17157450 | chr05 | 104384818 | 113.889.138 |
| rs1461227 | chr05 | 153149787 | 170.585.241 |
| rs11960137 | chr05 | 155270659 | 172.158.070 |
| rs1107154 | chr05 | 165319891 | 182.728.908 |
| rs11743823 | chr05 | 168097322 | 187.591.189 |
| rs6875659 | chr05 | 175091259 | 207.238.134 |
| rs860747 | chr06 | 14808928 | 31.902.943 |
| rs10498810 | chr06 | 56234862 | 88.103.229 |
| rs218862 | chr06 | 121425761 | 137.232.682 |
| rs10946113 | chr06 | 158555100 | 191.884.978 |
| rs12701745 | chr07 | 4365479 | 7.849.857 |
| rs7808899 | chr07 | 14586414 | 26.577.191 |
| rs2384982 | chr07 | 103300351 | 117.468.981 |
| rs2341823 | chr07 | 131551118 | 148.328.642 |
| rs10258063 | chr07 | 131285537 | 147.628.101 |
| rs2707575 | chr07 | 147076371 | 167.311.173 |
| rs1320892 | chr07 | 143189876 | 164.596.490 |
| rs17150066 | chr08 | 9347921 | 21.524.808 |
| rs2736340 | chr08 | 11381382 | 30.296.439 |
| rs956969 | chr08 | 62227592 | 91.125.498 |
| rs1373302 | chr08 | 73120270 | 101.784.532 |
| rs16880715 | chr08 | 111524781 | 133.174.536 |
| rs13280988 | chr08 | 112439692 | 133.487.375 |
| rs9642819 | chr08 | 114231199 | 133.948.808 |
| rs6994396 | chr08 | 114881166 | 134.253.422 |
| rs7017679 | chr08 | 115827888 | 134.530.637 |
| rs799889 | chr08 | 117320076 | 134.626.628 |
| rs2450219 | chr08 | 119372638 | 136.243.457 |
| rs10811305 | chr09 | 20047787 | 40.141.977 |
| rs12380601 | chr09 | 71878185 | 68.355.623 |
| rs1980889 | chr09 | 89128799 | 113.037.022 |
| rs3001115 | chr09 | 110483401 | 148.129.925 |
| rs10751867 | chr10 | 2339064 | 2.388.980 |
| rs3123687 | chr10 | 31569267 | 60.204.824 |
| rs2795918 | chr10 | 55847021 | 73.212.055 |
| rs17130385 | chr10 | 115186009 | 140.098.032 |
| rs1891760 | chr10 | 115249096 | 140.112.143 |
| rs1419138 | chr10 | 119721271 | 149.552.154 |
| rs9422913 | chr10 | 127335597 | 163.614.355 |
| rs874189 | chr11 | 15838275 | 30.146.467 |
| rs7927234 | chr11 | 27392932 | 49.491.567 |
| rs1396883 | chr11 | 34714023 | 66.734.662 |
| rs11034734 | chr11 | 38427247 | 72.051.190 |
| rs174570 | chr11 | 61353788 | 82.671.396 |
| rs7124676 | chr11 | 64069867 | 85.739.912 |
| rs1790740 | chr11 | 66886698 | 87.328.996 |
| rs568789 | chr11 | 83823790 | 109.549.426 |
| rs878874 | chr11 | 94228113 | 115.893.741 |
| rs485645 | chr11 | 125938189 | 163.994.520 |
| rs7103088 | chr11 | 129514516 | 170.216.645 |
| rs7961436 | chr12 | 10134132 | 24.480.403 |
| rs7958163 | chr12 | 112737962 | 136.354.954 |
| rs2585897 | chr13 | 20296979 | 1.650.505 |
| rs2151236 | chr13 | 40667660 | 40.773.991 |
| rs9576996 | chr13 | 39813272 | 39.772.371 |
| rs9533623 | chr13 | 43278753 | 43.829.229 |
| rs2078588 | chr13 | 71199633 | 74.381.570 |
| rs314580 | chr13 | 88709705 | 89.814.914 |
| rs9523747 | chr13 | 92095202 | 93.456.841 |
| rs1244379 | chr14 | 33750102 | 32.599.850 |
| rs2251244 | chr14 | 61093017 | 58.531.845 |
| rs10145908 | chr14 | 62823082 | 60.110.082 |
| rs6573746 | chr14 | 66576406 | 62.945.400 |
| rs7142344 | chr14 | 78319897 | 77.470.538 |
| rs7164838 | chr15 | 32754866 | 28.507.670 |
| rs8041147 | chr15 | 35085650 | 32.994.181 |
| rs8030587 | chr15 | 40769098 | 39.914.006 |
| rs2304580 | chr15 | 38816959 | 39.083.077 |
| rs735480 | chr15 | 42939663 | 40.303.854 |
| rs1453858 | chr15 | 46091402 | 43.468.063 |
| rs4646 | chr15 | 49290136 | 46.480.188 |
| rs2351254 | chr15 | 87381303 | 97.078.527 |
| rs4402506 | chr15 | 91931362 | 108.574.947 |
| rs7173885 | chr15 | 93608135 | 122.745.034 |
| rs4787040 | chr16 | 7500981 | 21.616.316 |
| rs764679 | chr16 | 8423521 | 26.003.869 |
| rs9923547 | chr16 | 12685472 | 34.399.526 |
| rs4791868 | chr17 | 9638969 | 25.702.315 |
| rs1074075 | chr17 | 48608764 | 93.954.865 |
| rs7211426 | chr17 | 51009547 | 96.701.310 |
| rs1369290 | chr18 | 65842500 | 106.372.948 |
| rs386569 | chr19 | 50478886 | 81.461.863 |
| rs4812381 | chr20 | 37420974 | 59.355.952 |
| rs718092 | chr20 | 37660430 | 59.657.466 |
| rs6101991 | chr20 | 38515722 | 64.910.078 |
| rs4812831 | chr20 | 42451674 | 70.731.407 |
| rs2836181 | chr21 | 38477680 | 45.212.340 |
| rs2837352 | chr21 | 40285648 | 49.403.948 |
| rs2187239 | chr21 | 42293624 | 56.467.707 |
| rs878825 | chr22 | 20306803 | 16.977.601 |
| rs1058552 | chr22 | 19386722 | 14.390.262 |
| rs132663 | chr22 | 34887524 | 45.563.474 |
| rs1557553 | chr22 | 43081521 | 57.180.376 |
