# Supplementary material for: Cuba: Exploring the History of Admixture and the Genetic Basis of Pigmentation Using Autosomal and Uniparental Markers
Source: PLoS Genet. 2014 Jul 24;10(7):e1004488. doi: 10.1371/journal.pgen.1004488 (PMC4109857; doi:10.1371/journal.pgen.1004488)
Supplement: Table S3 — SNPs on pigmentation genes. (DOCX) [file pgen.1004488.s009.docx]

| **Gene** | **Chromosome** | **SNP** | **Position** |
| --- | --- | --- | --- |
| *UGT1A* | 2 | rs6742078 | 234672639 |
| *SLC45A2* | 5 | rs35395 | 33948589 |
| *SLC45A2/MATP* | 5 | rs16891982 | 33951693 |
| *IRF4* | 6 | rs12203592 | 396321 |
| *TYRP1* | 9 | rs2733831 | 12703484 |
| *BNC2* | 9 | rs10756819 | 16858084 |
| *GATA3* | 10 | rs376397 | 8103298 |
| *GRM5 (linked to TYR)* | 11 | rs10831496 | 88557991 |
| *TYR* | 11 | rs1042602 | 88911696 |
| *KITLG* | 12 | rs642742 | 89299746 |
| *OCA2* | 15 | rs7495174 | 28344238 |
| *HERC2* | 15 | rs12913832 | 28365618 |
| *APBA2 (linked to OCA2)* | 15 | rs4424881 | 29261716 |
| *SLC24A5* | 15 | rs1426654 | 48426484 |
| *MC1R* | 16 | rs1805007 | 89986117 |
| *ASIP* | 20 | rs6058017 | 32856998 |

**Table S3.** SNPs on pigmentation genes.
