# Supplementary material for: Cuba: Exploring the History of Admixture and the Genetic Basis of Pigmentation Using Autosomal and Uniparental Markers
Source: PLoS Genet. 2014 Jul 24;10(7):e1004488. doi: 10.1371/journal.pgen.1004488 (PMC4109857; doi:10.1371/journal.pgen.1004488)
Supplement: Table S5 — Haplogroup assignations based on 18 mtDNA markers. (DOCX) [file pgen.1004488.s011.docx]

**Table S5.** Haplogroup assignations based on 18 mtDNA markers.

|  | **Haplogroups** | **Absolute Frequency** | **Relative Frequency** |
| --- | --- | --- | --- |
| **African** | L0/L1c1a2c/L2a1d1 | 28 | 3.0 |
|  | L0/L1c1a2c/L2a1d1/L1c6 | 1 | 0.1 |
|  | L1b1a3b | 1 | 0.1 |
|  | L3 | 139 | 14.8 |
|  | L3/N | 2 | 0.2 |
|  | L3'4 | 50 | 5.3 |
|  | L3'4'6 | 134 | 14.3 |
|  | L3d3/L3h1 | 9 | 1.0 |
|  | L4b1 | 1 | 0.1 |
| **Eurasian** | B5b1a | 1 | 0.1 |
|  | H | 70 | 7.5 |
|  | H1au1b/H5a1b/H7a/H27/H24b | 1 | 0.1 |
|  | H5a1b/H27 | 1 | 0.1 |
|  | J | 26 | 2.8 |
|  | J1b1a | 3 | 0.3 |
|  | JT | 28 | 3.0 |
|  | K1 | 5 | 0.5 |
|  | M | 9 | 1.0 |
|  | N | 21 | 2.2 |
|  | N1'5 | 1 | 0.1 |
|  | N1a1b | 7 | 0.7 |
|  | Non-R | 7 | 0.7 |
|  | R | 5 | 0.5 |
|  | R0 | 8 | 0.9 |
|  | U | 55 | 5.9 |
|  | X2 | 4 | 0.4 |
| **Native American** | A2 | 206 | 22 |
|  | A2i | 14 | 1.5 |
|  | B2 | 25 | 2.7 |
|  | C1 | 43 | 4.6 |
|  | D1 | 33 | 3.5 |
