# Supplementary material for: Cuba: Exploring the History of Admixture and the Genetic Basis of Pigmentation Using Autosomal and Uniparental Markers
Source: PLoS Genet. 2014 Jul 24;10(7):e1004488. doi: 10.1371/journal.pgen.1004488 (PMC4109857; doi:10.1371/journal.pgen.1004488)
Supplement: Table S6 — Contingency table analysis for mtDNA. (DOCX) [file pgen.1004488.s012.docx]

**Table S6.** Contingency table analysis using exact tests for mtDNA.

| **Provinces** | **European** | **%** | **African** | **%** | **Native American** | **%** |
| --- | --- | --- | --- | --- | --- | --- |
| **PR** | 33 ^a^ | 49.3 | 25 | 37.3 | 9 ^b^ | 13.4 |
| **AR** | 20 ^a^ | 52.6 | 11 | 28.9 | 7 ^b^ | 18.4 |
| **MY** | 14 ^a^ | 43.8 | 13 | 40.6 | 5 ^b^ | 15.6 |
| **LH** | 31 | 32.6 | 42 | 44.2 | 22 ^b^ | 23.2 |
| **MT** | 41 ^a^ | 57.7 | 21 | 29.6 | 9 ^b^ | 12.7 |
| **CF** | 14 | 36.8 | 19 | 50.0 | 5 ^b^ | 13.2 |
| **VC** | 19 | 29.2 | 18 | 27.7 | 28 | 43.1 |
| **SS** | 9 | 21.4 | 17 | 40.5 | 16 | 38.1 |
| **CA** | 14 | 34.1 | 10 | 24.4 | 17 | 41.5 |
| **CG** | 17 | 24.6 | 26 | 37.7 | 26 | 37.7 |
| **LT** | 10 | 20.8 | 10 ^b^ | 20.8 | 28 ^a^ | 58.3 |
| **HG** | 8 ^b^ | 7.5 | 35 | 33.0 | 63 ^a^ | 59.4 |
| **GR** | 5 ^b^ | 7.2 | 36 ^a^ | 52.2 | 28 | 40.6 |
| **SC** | 6 ^b^ | 6.4 | 54^a^ | 57.4 | 34 | 36.2 |
| **GT** | 10 | 18.5 | 25 | 46.3 | 19 | 35.2 |
| **IJ** | 1 | 11.1 | 3 | 33.3 | 5 | 55.6 |
| **Chi-Square** | P=0.0000 | | P=0.0000 | | P=0.0000 | |

Provinces with superscripts ^a&b^, the ancestral maternal contributions are statistically significant different (a=higher, b=lower) than the rest of the provinces.
