# Supplementary material for: Cuba: Exploring the History of Admixture and the Genetic Basis of Pigmentation Using Autosomal and Uniparental Markers
Source: PLoS Genet. 2014 Jul 24;10(7):e1004488. doi: 10.1371/journal.pgen.1004488 (PMC4109857; doi:10.1371/journal.pgen.1004488)
Supplement: Table S7 — Haplogroup assignations based on Y-chromosome markers. (DOCX) [file pgen.1004488.s013.docx]

**Table S7.** Haplogroup assignations based on Y-Chromosome markers.

| **Ancestry** | **Y-Chromosome**  **Haplogroups** | **Absolute Frequency** | **Relative Frequency** |
| --- | --- | --- | --- |
| **African** | BT | 68 | 17.7 |
| **Eurasian** | F | 96 | 25.0 |
|  | K | 4 | 1.0 |
|  | R1a1 | 4 | 1.0 |
|  | R1b1a2 | 169 | 44.0 |
|  | T1a | 1 | 0.3 |
|  | R1b1b2a1 | 40 | 10.4 |
| **Native American** | Q1a2a1a1 | 2 | 0.5 |
