# Supplementary material for: Cuba: Exploring the History of Admixture and the Genetic Basis of Pigmentation Using Autosomal and Uniparental Markers
Source: PLoS Genet. 2014 Jul 24;10(7):e1004488. doi: 10.1371/journal.pgen.1004488 (PMC4109857; doi:10.1371/journal.pgen.1004488)
Supplement: Table S8 — Contingency table analysis for Y-chromosome. (DOCX) [file pgen.1004488.s014.docx]

**Table S8.** Contingency table analysis for Y-chromosome.

| **Provinces** | **Eurasian** | **%** | **African** | **%** | **Native American** | **%** |
| --- | --- | --- | --- | --- | --- | --- |
| **PR** | 21 | 91.3 | 2 | 8.7 |  | 0 |
| **AR** | 10 | 83.3 | 2 | 16.7 |  | 0 |
| **MY** | 11 | 84.6 | 2 | 15.4 |  | 0 |
| **LH** | 35 | 83.3 | 7 | 16.7 |  | 0 |
| **MT** | 26 | 92.9 | 2 | 7.1 |  | 0 |
| **CF** | 16 | 76.2 | 5 | 23.8 |  | 0 |
| **VC** | 26 | 70.3 | 10 ^a^ | 29.7 |  | 0 |
| **SS** | 15 | 88.2 | 2 | 11.8 |  | 0 |
| **CA** | 10 | 100 | 0 | 0 |  | 0 |
| **CG** | 32 | 76.2 | 8 | 21.4 | 1 | 2.4 |
| **LT** | 17 | 85.0 | 0 | 15.0 |  | 0 |
| **HG** | 37 | 82.2 | 7 | 17.8 |  | 0 |
| **GR** | 18 | 90.0 | 2 | 10.0 |  | 0 |
| **SC** | 21 ^b^ | 63.6 | 11 ^a^ | 33.3 | 1 ^a^ | 3.0 |
| **GT** | 16 | 94.1 | 1 | 5.9 |  | 0 |
| **IJ** | 3 | 75.0 | 1 | 25.0 |  | 0 |

Provinces with superscripts ^a&b^, the ancestral maternal contributions are statistically significant different (a=higher, b=lower) than the rest of the provinces.
